# Vocal Behavior of Teachers Reading with Raised Voice in a Noisy Environment

**DOI:** 10.3390/ijerph19158929

**Published:** 2022-07-22

**Authors:** Manfred Nusseck, Anna Immerz, Bernhard Richter, Louisa Traser

**Affiliations:** Freiburg Institute for Musicians’ Medicine, Medical Faculty of the Albert-Ludwigs—University Freiburg, University of Music Freiburg, 79110 Freiburg, Germany; anna.immerz@uniklinik-freiburg.de (A.I.); bernhard.richter@uniklinik-freiburg.de (B.R.); louisa.traser@uniklinik-freiburg.de (L.T.)

**Keywords:** teachers’ voice, vocal health, phonation time, Lombard effect

## Abstract

(1) Objective: Teaching is a particularly voice-demanding occupation. Voice training provided during teachers’ education is often insufficient and thus teachers are at risk of developing voice disorders. Vocal demands during teaching are not only characterized by speaking for long durations but also by speaking in noisy environments. This provokes the so-called Lombard effect, which intuitively leads to an increase in voice intensity, pitch and phonation time in laboratory studies. However, this effect has not been thoroughly investigated in realistic teaching scenarios. (2) Methods: This study thus examined how 13 experienced, but vocally untrained, teachers behaved when reading in a noisy compared to quiet background environment. The quiet and noisy conditions were provided by a live audience either listening quietly or making noise by talking to each other. By using a portable voice accumulator, the fundamental frequency, sound pressure level of the voice and the noise as well as the phonation time were recorded in both conditions. (3) Results: The results showed that the teachers mainly responded according to the Lombard effect. In addition, analysis of phonation time revealed that they failed to increase inhalation time and appeared to lose articulation through the shortening of voiceless consonants in the noisy condition. (4) Conclusions: The teachers demonstrated vocally demanding behavior when speaking in the noisy condition, which can lead to vocal fatigue and cause dysphonia. The findings underline the necessity for specific voice training in teachers’ education, and the content of such training is discussed in light of the results.

## 1. Introduction

For occupational voice users, the voice is an important tool for the successful practice of their profession [1]. A particularly voice-demanding profession is that of classroom teachers. The amount of voice-use during a school day is characterized by instructions and explanations to impart knowledge, establish classroom management, and to develop a relationship with the class. It has been shown that teachers speak more during work than during leisure time [2]. Teaching therefore involves various vocal risk factors [3,4] which may cause the development of dysphonia [5,6]. The duration of speaking is not the only significant risk factor as speech volume also plays an important role: teachers were found to talk with a raised voice 61% of the time during actual classroom lessons [7]. Thus, in addition to the quantitative factor of prolonged speaking duration, elevation of sound pressure level is also a factor worth considering in regard to the occupational vocal load of teachers.

Teachers are at higher risk of developing pathological vocal symptoms [8]. A large number of publications found that teachers report voice problems significantly more often than other occupational groups [9,10,11,12]. This includes vocal discomfort, decreased voice quality and vocal endurance [13]. A meta-analysis found that female gender, additional airway problems, high caffeine consumption and higher number of classes per week were additional risk factors for voice problems among teachers [14]. Further vocal demands can also be caused by psychological stress [15], size and discipline problems in the class [16], as well as poor indoor air quality in the classroom [17].

These occupational voice risk factors are important not only in terms of teachers’ health, but also concerning the quality of teaching. Studies showed that teaching with a stressed voice, i.e., recognizably hoarse and rough, led to lower learning outcomes in pupils compared to teaching with a clear voice [18,19,20,21]. Findings suggest that children listening to a stressed voice require certain cognitive capacities to process the speech signal at the expense of comprehension [22]. Furthermore, background noise in connection with a poor voice quality of the teacher degrades listening abilities in classrooms drastically [23].

Regardless of how long teachers speak during class, which depends strongly on the lesson subject, students’ age, and the kind of teaching method [7], it is of particular interest to understand why teachers raise their voice and what happens in these instances. One reason to increase voice intensity is when speaking in noisy environments. These situations trigger adaptations in the speaker’s voice production, which are commonly referred to as the Lombard effect [24]. Speech production in noise is therefore called Lombard speech, whereby specific voice modifications in accordance with the noise source aim to facilitate intelligibility and maintain comprehension in communicative situations. 

A review article showed that numerous studies have investigated vocal changes during Lombard speech [25]. The most common changes found in healthy, vocally untrained subjects describe adaptations of increasing the voice sound pressure level (SPL) and fundamental frequency (ƒ_o_) [26,27,28] as well as additional effects on the acoustic properties of voice production, such as pronounced amplitude modulations [29] and increases in duration and intensity of vowel production [27,30]. In particular, changes in the duration of vowels and voiced consonants were found to be typical for Lombard speech [24,31,32]. Generally, lengthened vocalizations in noisy environments were found to be interrelated with the voice SPL, particularly on stressed words [24]. Similarly, ƒ_o_ and the duration of words were also found to be correlated [33]. However, this may be caused by rhetorical pronunciations where words are intentionally modulated for prosaic expression. When speaking in noisy environments with generally raised voice instead of just specific words, the increased intensity contour of the voice was found to be equal across all words in the utterance [34]. Nevertheless, while the duration of vowels increases, the duration of unvoiced stops and fricatives, i.e., voiceless consonants, decreased [28,35]. 

In addition to the acoustic parameters of the voice, specific respiratory strategies also change when speaking in noisy environments. During speaking, respiratory kinematics are characterized by short and fast inhalations while voicing is produced on prolonged exhalation [36]. The depth and duration of the breathing is rather irregular and depends on the utterance produced. When reading aloud, the inhalation is synchronized with the linguistic structure of the text. Speakers take breaths related to syntactic components such as the end of a sentence or a paragraph. When investigating the lung volume variability during reading, high consistencies in the location of inspirations across participants were found at grammatically appropriate places in the texts [37]. Increased inhalation duration and volume have been observed, especially associated with louder utterances, at sentence and paragraph boundaries, and in the initial breath before the reading [38].

Studies investigating vocal behavior in noisy environments were usually performed in experimental settings, i.e., laboratories or sound studios, where speakers were recorded with and without masking noise [39,40]. Very few studies used realistic environments to analyze Lombard speech. For instance, Patel and Schell [34] examined speech production during a cooperative game in which two people interacted with each other verbally in a quiet or a noisy setting. The authors found that the speakers proportionally increased voice intensity, ƒ_o_ and word duration in accordance with the noise level. However, in this scenario only game-related words in spontaneous speech were analyzed.

Investigating Lombard speech in experimental and clinical settings does not necessarily represent the environments of occupational voice users. For instance, in previous studies, 85 dB broad band noise has been used to imitate a noisy environment [26,27,28]. This is in contrast to the typical classroom background noise of about 65–70 dB [7,41,42]. The acoustical characteristics of noise in a classroom would also be better characterized by the acoustic properties of cocktail party noise than broadband white noise. Former studies also often used specific words or single sentences for acoustical evaluation, and yet, it is of great importance to understand the normal and healthy function of the voice in a typical work environment [8].

To investigate the vocal demand responses of teachers during a working day, several studies have used a portable voice dosimeter to record both voice and background noise. This device is a voice accumulator with a lightweight neck collar carrying a microphone and an accelerometer. It switches between measuring the ambient noise and the person’s own voice, depending on whether or not the person phonates. It thus allows the monitoring of the ƒ_o_, SPL and phonation time during phonation. SPL is recorded for the vocal intensity and the background noise. This technique was used to evaluate vocal behavior in realistic environments over a longer period. Vocal SPL, ƒ_o_ and the phonation time were found to be significantly higher in teaching situations compared to non-teaching situations [2,43,44,45] confirming that teachers experience extensive vocal demands at work. Analysing the vocal behavior of teachers during classroom lessons with a voice dosimeter, teachers showed rather individual vocal demand responses according to the subject being taught, with the highest voice intensities in sports lessons [42]. While the noise level varied considerably during the lesson and was loudest at the beginning and at the end of a lesson, the SPL of the voice varied less within the lessons.

Speaking in noisy environments has been shown to cause voice problems and can lead to chronic voice disorders [46]. Intensifying voice production is associated with an increase in subglottic pressure, airflow over the glottis and maximum airflow declination rate [47,48] and also an increase in the mechanical impact stress of the vocal folds [49]. This has been considered as a key factor to the ethology of voice disorders, including secondary organic vocal fold lesions such as nodules or polyps [50]. Therefore, this relationship indicates that a prolonged period of Lombard speech is a potential risk factor for the development of voice disorders. This was shown in a study with individuals diagnosed with phonotrauma who experienced higher environmental noise levels in their daily life than individuals diagnosed with a functional voice disorder [51]. 

Based on their research findings, there is a call for the urgent promotion of occupational voice health [8] and strong recommendations for the general implementation of vocal education for teachers [6,52]. This is consistent with other studies calling for more research on vocally demanding occupations and environmental noise contributing to vocal health and preventing occupational voice disorders [46,53]. There is evidence that voice intervention programs can have positive impacts on voice quality in short-term [54,55,56,57] and even in long-term perspective [58]. While the effects of Lombard speech have been studied before and after the vocal effort of a typical teachers’ workday [45], there is still a lack of knowledge regarding the intuitive compensatory strategy of teachers during the Lombard speech episode itself in a realistic setting. Individualized training programs, which are adapted in detail to the vocal demands of teachers, should concretely expound these possibly uneconomical compensation strategies being used by teachers intuitively during Lombard speech. 

The goal of this study was to investigate the impact of environmental noise on the voice production of teachers within a realistic setting. The participants were asked to read a text aloud in a quiet and a noisy condition. The voice was recorded using a voice dosimeter, which can provide an authentic picture of vocal demand responses [59]. In contrast to laboratory situations, this study was performed in a semi-controlled real situation with a live audience and voice changes were investigated. To achieve this, rooms very similar in size and acoustics to a typical classroom were used, with people sitting at tables and the speaker standing in front of them. The audience was either quietly listening or chatting with each other. The latter simulates a common classroom situation where, for example, students work in small groups, and especially at the beginning and at the end of a lesson [42].

According to the literature, it was expected that voice SPL, ƒ_o_ and word durations would increase during Lombard speech. In addition to these parameters, of particular interest was the phonation time, the voicing, and inhalation duration across both conditions. It was assumed that the phonation time would also increase in the noisy condition due to a lengthening of the words. However, it was also expected that the pauses between words would shorten and the duration for inhaling would increase due to a greater subglottic air-pressure being needed during loud reading. The results of this study might help to further understanding of physiological variables that influence vocal demand responses of occupational voice users, and thus allow further insight into the pathophysiology and the prevention of voice disorders associated with Lombard speech.

## 2. Materials and Methods

### 2.1. Participants

The sample of this study included 13 high school teachers (7 female and 6 male). The mean age was 52.0 years (SD 10.5 years) without significant difference between genders. They were participants of voice workshops with group sizes between 8 and 13 persons. In each of the workshop, two persons (one female, one male) were asked to participate in the experiment. After explaining the procedure and the measuring technique, they volunteered for participation. The experiment was performed at the beginning of the workshops. At this point, the participants were not yet influenced by the content of the workshop. They also had not taught or worked before the workshop so that they were not vocally tired. After the experiment, the individual results of the participants were discussed in the workshop.

Except for the gender and the age, no further information was gathered from the participants. The study was included in a larger project of teacher’s vocal health [55] and within this project, the study was approved by the Ethics Committee of the University Clinic Freiburg.

### 2.2. Procedure

The participants read an unknown text aloud in front of the workshop group. While the audience was sitting at tables, the speakers were standing in front of them. The audience consisted of the other workshop participants, i.e., 7–12 persons roughly evenly divided in genders. The study was conducted in three different rooms. The sizes and the acoustics of the rooms were rather similar and resembled a typical classroom for a size of about 25 students. Reverberation times were not measured. 

In the first condition (Quiet situation), the speakers were asked to read the first part of the text in a usual manner of reading a text to an audience (i.e., a school class). The other participants in the group listened silently. In the second condition (Noisy situation), the audience produced some background noise and the participants read the second part of the text. To produce a standardized noisy environment that is similar to people chatting, the audience was asked to speak a single word (“Rhubarb”) continuously to each other with a vocal intensity of telling a story at close distance. The use of a single word comes from early movies. To simulate a realistic babble of voices, actors had to constantly repeat the word “Rhabarber”.

The speakers had to read the text with the intention of maintaining intelligibility above the background noise. This represents a demanding classroom situation when the teacher attempts to reach the students when they are noisy and active. To do this, the teacher tries to calm down the class and make them quiet. Generally, it does not take long to get the class quiet. However, the vocal demands response of the teachers in such situations is very similar to the noisy condition of this study.

All speakers performed first the quiet condition and then the noisy condition. There was a little pause in between for instructing the audience. The performance lasted about 5 min per person including the installation of the voice-measuring device. Since a loud speech might lead to vocal fatigue, it was assumed that speaking in a silent situation at normal voice intensity would cause only low vocal strain and therefore minimize the degree of fatigue. In addition, all speakers were perceived as vocally healthy teachers by the leader of the workshop. They also did not verbally report any vocal symptoms. This supported the assumption of low vocal demands for the participants in the quiet situation. Other health related issues were not collected from the participants.

### 2.3. The Text

The chosen text for the experiment was a German text about music in the Middle Ages. It was divided into two parts, one for each condition. Both were nearly identical from a phonological point of view. The first part contains 118 words with 876 characters (including spaces) with 46% monosyllabic words (1 syllable) and 31% polysyllabic words (≥3 syllables). The mean syllables per word is 2.1 and the mean number of characters per word is 6.4. The passage has 38% vowels and 30% voiced consonants (sonorants). The second part contains 166 words with 1150 characters (including spaces). This part also includes 45% monosyllabic words and 23% polysyllabic words. Similar to the first part, the mean syllables per word is 1.9 and the mean number of characters per word is 6.0 and it contains 38% vowels and 31% voiced consonants. With approximately 1% of the characters being punctuation marks, both texts consist of 30% voiceless consonants. Since the number of syllables per word and the number of vowels and consonants are very similar in both parts, it can be assumed that both parts are phonetically the same.

All participants read the text in a very straightforward manner, i.e., without long pauses or interruptions. They did not perform with any emphasized phrasings or accentuations that might have caused longer duration of vowels [60]. The average reading time across all participants was 57.6 s (SD 13.5 s) for the first part and 72.3 s (SD 14.4 s) for the second part.

### 2.4. Measuring Technique

The portable dosimeter VoxLog (Sonvox, Umea, Sweden) was used. The device is a lightweight neck collar with a microphone and an accelerometer. The microphone records the sound of the environment. The accelerometer sensor detects when the person phonates, labelling this as voice data. When the person did not speak, the recording was saved as ambient noise data. The recording frequency was set to 0.1 s. 

With the software of the device (“VoxLog Connect 3.1.13”, Levis, QC, Canada), the ƒ_o_ and the SPL of the voice and the noise were collected. The device has previously been successfully used to collect voice data of teachers in natural environments [42,44,61,62,63]. 

The accelerometer records speech data only when the subject is producing sounds via vocal fold vibration (e.g., phonation of vowels and voiced consonants). During this time, the value of the noise was set to zero. In the analysis, all noise values with zero scores were excluded for calculating the mean noise SPL. Similarly, the mean voice SPL was also calculated only when the voice value was not zero.

The phonation and the pausing times were analyzed from the voice data. The phonation time was averaged within the sampling frequency and reported in % of the recorded time frame. The times of no phonation were classed as pausing times. Since a text was constantly read aloud, the moments of no phonation were either when the person was inhaling or at little pauses between words. Due to the sampling frequency, very short voiceless consonants were included in the phonation time. Only pauses of at least 100 ms were recorded as non-phonation time.

In previous studies, the mean inhalation duration was found to be 0.54 s (SD 0.18 s) with a range from a minimum of 0.19 s to a maximum of 1.21 s during a reading task [64]. Therefore, pauses >200 ms were considered to be inhalation durations. Very long pauses (>2 s) were also excluded as they might contain more breaths or other behaviors such as swallowing. Therefore, three voice conditions have been analyzed: the mean durations of (1) the voicing, (2) the short pauses, and (3) the long pauses including inhalation.

The analysis of the recordings started with the first phonated word of the speaker. Therefore, it did not include the initial inhalation before the reading. The measurements also ended with the last spoken word. Therefore, the analysis was only performed on the pure reading time.

### 2.5. Statistics

The statistical analysis was performed with SPSS 28 (SPSS Inc., Armonk, NY, USA). Descriptive statistics were reported with mean values and standard deviations (SD). Comparisons of parametric variables between the conditions were performed with paired sample t-tests. Influences of further factors such as gender were investigated using univariate repeated measures ANOVA. Non-parametric comparisons were analyzed with a cross-table and Pearson’s Chi^2^ was reported. Correlations between two parametric variables were described with the coefficient Pearson’s r. The level of significance was set to *p* = 0.05.

## 3. Results

The mean values of the vocal parameters in both the quiet and the noisy condition are shown in Table 1. The fundamental frequencies ƒ_o_ has been separately analyzed by gender. All values significantly increased in the noisy condition compared to the quiet condition. While the noise SPL increased by about 11.4 dB(A), the voice SPL increased by 8.4 dB(A). There were no significant interaction effects of gender for changes in voice SPL or the phonation time.

**Figure 1:**
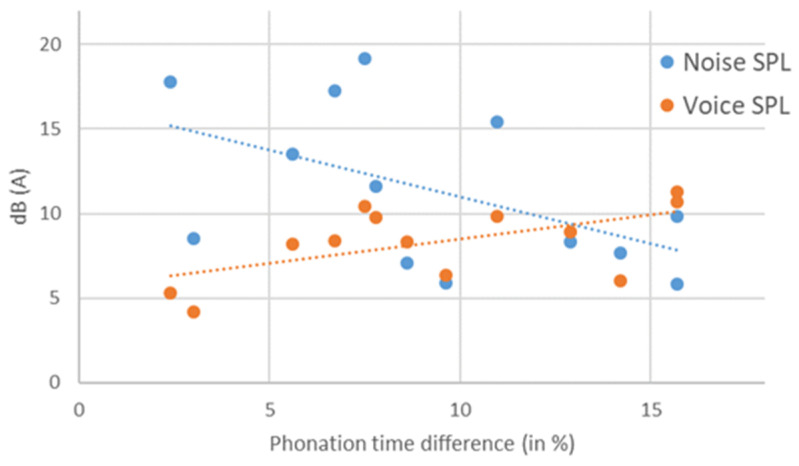
Increase in the phonation time of the voice showed a significant correlation with increases in sound pressure level (SPL) of the voice but not the noise. The figure displays differences in the voice SPL (orange) and the noise SPL (blue) between the conditions against the difference of the phonation time.

In both the quiet and the noisy condition, there were no significant correlations between voice SPL, noise SPL and phonation time. The correlations of the SPL difference between the conditions of the voice and the noise with the phonation time difference are shown in Figure 1. Increases in the voice SPL showed a significant correlation with increases in the phonation time (r = 0.58, *p* = 0.039). The noise SPL difference showed no significant correlation with the phonation time differences (r = −0.52, *p* = 0.071). The voice SPL difference and the noise SPL difference were not significantly correlated (r = 0.10; *p* = 0.741).

**Figure 2:**
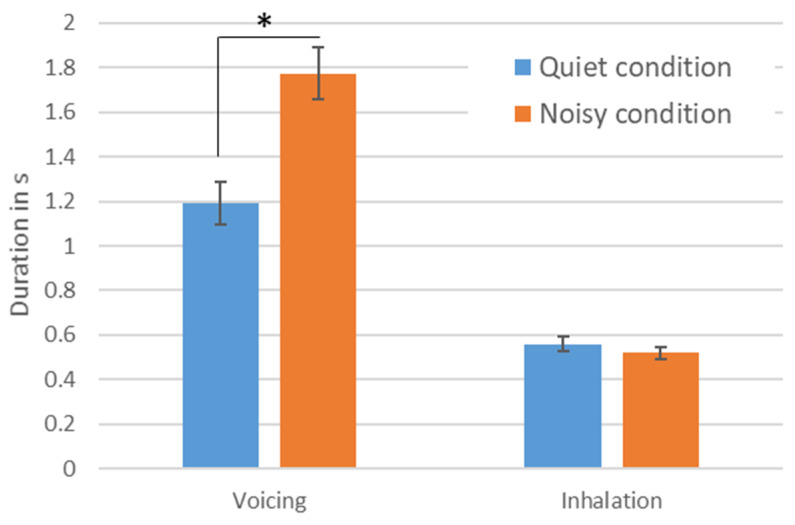
Significant increase in duration of phonation from quiet to noisy condition with stable duration of inhalations. The figure displays Mean durations of the voicing and the pauses (>200 ms) between conditions (error bars: standard error of the mean; *: *p* < 0.01).

The mean durations of the voicing and the inhalation pauses (>200 ms) are shown in Figure 2. The voicing durations increased significantly from 1.19 s (SD 0.33 s) in the quiet condition to 1.77 s (SD 0.08 s) in the noisy condition (t(12) = 7.58, *p* < 0.001). The duration of the inhalation pauses were not significantly different between the conditions (t(12) = 1.55, *p* = 0.148), with an average of 561 ms (SD 110 ms) in the quiet condition and 521 ms (SD 96 ms) in the noisy condition. The mean durations were very similar to other studies [38]. For both voicing and inhalation durations, there were no significant interaction effects found between genders.

The short pause durations were divided into two groups with pauses of 100 ms and 200 ms. For both groups, the number of pause occurrences was counted. Table 2 shows these numbers and the percentage of the total amount of occurrences in the quiet and the noisy conditions. The distribution of the pause occurrences between the two conditions was significantly different (Chi^2^ = 3.99, *p* = 0.046).

## 4. Discussion

In this study, the vocal demand responses [13] of teachers in a realistic environment when reading a text aloud in front of an audience were investigated in a quiet and a noisy background condition. For occupational voice users such as teachers, high noise levels during work require specific vocal behaviors. The results showed that the teachers increased ƒ_o_, voice SPL and phonation time in the noisy condition. At first glance, the teachers’ vocal performance in this realistic scenario was similar to the findings of previous studies in experimental and clinical settings. However, some specific differences were noted.

With an increase in the ambient noise, an increase in vocal intensity was observed, which is typical in Lombard speech. Nevertheless, no significant correlation was found between the changes in the voice SPL and the changes in the noise SPL. This suggests that the increase in vocal intensity was rather independent of the ambient noise level. It seems that it did not matter how much the noise level increased, the teachers raised their voice to a certain individual level. It is possible that they were performing as they would usually do in a classroom. This finding indicates that teachers who regularly raise their voice during work may adapt to the vocal demands by adopting an individual vocal effort of increasing the voice SPL to a rather high level. 

Speaking with increased voice SPL is a risk factor for impairing voice quality due to an increase in vocal fold collision stress, which strongly depends on the vocal fold velocity at the time of impact [65]. Additionally vocal fold impact stress correlates positively with subglottic pressure which in turn is also closely related to vocal intensity. Such mechanical stress has been considered as a key factor to the ethology of voice disorders including secondary organic focal fold lesions such as nodules or polyps [50]. Therefore, the behavior of the teachers in increasing the voice SPL independently of the noise creates an unnecessary amount of vocal fatigue. This can be avoided if the vocal effort of increasing the voice SPL fits the situational increase in the ambient noise SPL only to the degree of maintaining the intelligibility and reduces with a decreasing of the noise level. Former studies showed that teachers reacted rather differently to noise. While some teachers adapted their voice intensity to the noise level, others did not [41,42]. It is therefore necessary to not only train teachers in how to raise their voice, but also in how to adapt their voice appropriately to the situation.

Another possible vocal risk factor is to speak with increased ƒ_o_. It was also found in the present study that a noisy speaking condition caused an intuitive increase in ƒ_o_ of the teachers by about a half-semitone per 1 dB increase in SPL, which is in accordance with the literature in terms of magnitude [66]. This is typical in Lombard speech but also generally for louder speaking phonation [67]. Whereas in previous studies this increase was mainly attributed to an increase in subglottic pressure, which would passively raise ƒ_o_ [66], it was later described to be used in an active manner to increase vocal intensity as it results in a larger number of speech pressure cycles per time unit which also raises SPL [67]. Still, an increase in pitch would not only be related to a higher number of vibratory cycles and subglottic pressure but also greater acceleration of the vocal folds and thus higher inertia- as well as collision-forces which might correlate with a greater load for the vocal folds [68]. In one study, voice changes in 22 female teachers were measured before and after a typical workday, and it was found that some teachers showed an increased ƒ_o_ at the end of the day while the ƒ_o_ decreased in others [45]. The authors interpreted the increased ƒ_o_ as an adaptation of the voice due to the vocal demands and the decreased ƒ_o_ as an acute inflammation or muscle fatigue. It was also found that vocally trained speakers were able to maintain ƒ_o_ when speaking in noisy environments with increased voice SPL [69]. This might reflect better vocal control as the result of vocal training, which might also reduce the amount of vocal fatigue. However, the increase in the ƒ_o_ for loud phonation was not only shown for untrained voice users but also for singers and actresses [66,70] who, because of their training, should not behave in a fundamentally uneconomic way. However, it is not unlikely that singers, for example, choose appropriate resonance strategies according to their genre (anti-megaphone vocal tract for classical singers or megaphone-shaped vocal tract for contemporary music singer [71,72]) for higher phonation, which could additionally support the vocal folds in their vibration due to source–filter interactions. On the other hand, it could be that untrained subjects achieve the increase in pitch in a more uneconomic way (e.g., laryngeal elevation, which was associated with muscle tension dysphonia [73]). Thus, it could be concluded that a certain increase in pitch might be advantageous for loud speech, although the right amount and the realisation certainly plays an important role here, meaning that the negative properties in terms of increased mechanical stress do not predominate. Still, mean speaking pitch was found to be lower for trained voices compared to untrained participants [69] and professional broadcasters, for example, who often use a lower speaking pitch which was also found to arouse a greater sense of credibility, trust, and confidence, which could also be an important factor for teachers [74]. 

Furthermore, the performed correlation analysis showed a clear relationship between the increase in vocal intensity and the increase in phonation time. The participant not only spoke louder, but, proportionally, they also spoke for longer. The detailed analysis yielded that they mainly increased the duration of the vocalisation, i.e., the duration of vowels and voiced consonants. This finding is in line with previous studies [24,27,30,32]. With an increase of 12 dB(A) of the voice SPL, it was found that vowels were significantly lengthened by 33 ms on average [28]. The results showed that the voice SPL of the teachers increased by about 8.4 dB(A) and the mean voicing duration increased by 58 ms. The stretching of the voiced words in this study was therefore slightly longer than in Garnier and Henrich [28], but also shorter than the word duration increase of 77 ms found in Pittman and Wiley [31]. These findings indicate that the longer phonation time was mainly caused by the lengthening of the voiced parts of the words.

Nevertheless, stretching the voicing of words while speaking can sound like an artificially exaggerated lengthening. To avoid that and to keep a rather similar speaking pace, a possible strategy would be to shorten the soundless parts. One particular voiceless part is the pause to inhale. A study showed that during a reading task in German, there was a significant difference in inhalation duration according to the length of the sentence, but not regarding the syntactic complexity [38]. They found nearly identical pause durations in complex and simple sentences (548 ms and 540 ms, respectively). These durations were very similar to the inhalation durations found in this study. The duration of 561 ms in the quiet condition was even slightly longer than in the noisy condition with 521 ms.

Thus, the results showed that the durations of inhalation were not significantly different between the quiet and the noisy condition. The teachers inhaled with rather similar speed in both conditions. As the duration of inhalation is strongly correlated with the inhaled lung volume, it can be assumed that the teachers also inhaled approximately the same lung volume for phonation in both the quiet and noisy conditions. This might be another indication of uneconomic vocal behavior which can increase vocal fatigue when speaking louder: An increase in vocal intensity is frequently associated with an increase in subglottic pressure [75]. As deeper inhalation generates higher subglottic pressures through activation of a greater amount of passive recoil forces [76], it is believed to be a meaningful adaptation for loud phonation. The inspiratory airflow duration and the value for the inhaled volume of professional speakers during loud speaking were found to be significantly higher compared to untrained speakers [69]. The authors claimed that this is related to better management of breathing function due to years of voice training. Moreover, another study observed that professional speakers used higher lung volumes and greater volume excursions when performing monologs compared to conversational speech [77]. Conversely, phonation on low lung volume was more often associated with pressed phonation and voice disorders [78]. To avoid increasing duration and thus volume of inhalation during loud phonation means that the increase in subglottic pressure was not generated via passive recoil forces but might be related to changes in the vibratory pattern, which could be associated with higher adduction and thus higher mechanical stress during vocal fold vibration. Additionally, deeper inhalation should be associated with higher diaphragm activity which is closely related to the amount of tracheal pull [79]. A greater tracheal pull can affect the vertical laryngeal position in a caudal direction and increase vocal fold abduction, both of which are frequently desired in the function of the speaking voice [80,81]. It is therefore recommended to intentionally take more time to inhale more deeply when speaking louder to avoid an increase in mechanical impact stress on the vocal folds due to pressed phonation.

Other studies have found that the duration of voiceless consonants decreases when spoken louder [28,35]. This was supported by the findings of the teachers in this study. The distribution of the short pauses with 100 ms and 200 ms were significantly different between the quiet and the noisy condition. The distributions showed that there were more 200 ms pauses in the noisy condition than in the quiet condition. However, considering that the two sections of text are very similar in terms of the number of vowels, and voiced and unvoiced consonants, the total number of short pauses should be quite similar. This was not the case. There were fewer short pauses of 100 ms in the noisy condition than in the quiet condition but a rather similar number of 200 ms pauses. It can therefore be assumed that the speakers shortened many unvoiced consonants below the sampling frequency, which were not detected. It has been found that voiceless consonants were significantly shortened by 10 ms on average [28]. The analysis therefore suggests that the teachers did shorten the voiceless pauses and consonants. As an outcome, this can lead to unclear and blurred pronunciation and a loss of clear articulation, which can impair intelligibility and thus the need for increased SPL. It is therefore recommended to keep the articulation as clear as possible especially when speaking with a raised voice.

A study with trained and untrained singers found that untrained participants overexerted their vocal system after a duration of loud speaking and began to show strong signs of vocal fatigue [82]. In contrast, the trained participants were able to use their knowledge and abilities to maintain vocal health during intensive speaking. Therefore, vocal training focusing on speaking with a raised voice is highly recommended for teachers. The studies with professional speakers in particular demonstrate that voice training can reduce vocal effort and maintain long term vocal health [69,77].

To summarize, the teachers generally adapted their voices in a noisy environment according to previous findings considering Lombard speech. In contrast to the fact that the subjects were highly occupational voice users, they still exhibited some behaviors which could rather be attributed to an uneconomic voice production (see Table 3). Therefore, it can be seen as essential to provide some specific recommendations for teachers who are required to raise their voices. Five particular features of voicing in noise have been elicited from this study. These are: increases of the voice SPL, the ƒ_o_ and the voicing duration, as well as the similar inhalation duration and the shortening of voiceless consonants in a noisy environment compared to a quiet environment. These aspects are listed in Table 3 along with the potential risk factors and some recommendations on how to avoid vocal fatigue. 

The recommendations are common methods from voice therapy and voice training. To implement these recommendations, certain exercises can be used. These are very individual and require contact with a professional voice coach. 

It was found that individuals diagnosed with phonotrauma spoke more in environments with higher noise levels than individuals with functional voice symptoms [51]. However, it is still unclear how much the noisy surrounding really caused the vocal impairment. Nevertheless, during a classroom lesson, there are two types of sound which compete: the noise of the class and the voice of the teacher. While studies recommend reducing noise in the classroom, this may not be practical in some situations. Since the voice can withstand higher intensity for short durations, it would make sense to train the voice for such circumstances. From a didactical perspective, it would be advisable to use the trained voice to reduce the noise during class and create an optimal and quiet learning atmosphere. 

### Limitations

As recommended by several studies, research on vocal demand responses in realistic environments is necessary. However, this requires special measurement procedures and a corresponding realistic situation. Such studies are quite extensive and take time to conduct. For these reasons, the sample size of this study was rather small. It is necessary to conduct further studies in classroom environments with more participants to obtain more detailed data and findings.

For that, it would be recommended to measure other acoustic variables of the rooms as well, such as the reverberation time. This might have an influence on the vocal demand response. In this study, typical classrooms were chosen to simulate the usual situation.

Regarding the sample, it would have been interesting to collect more details of the participants such as general health, vocal habits, and vocal symptoms. However, this would require a larger sample size to provide the possibility of selecting groups according to their background.

Furthermore, the recording device of the portable dosimeter was limited to a rather low sampling frequency and did not include spectral measures. However, an increase in SPL and perceived loudness during speaking phonation might also be related to a change in vocal tract articulation and thus resonance properties of the vocal tract (e.g., resonance maxima in the frequency region where our ear is most sensitive, as with the speakers’ formant [83]) as well as interactions between the vocal tract and the vocal fold vibrations [83]. Differences in supraglottal adaptions were shown, for example, in an endoscopic study between actors and untrained subjects [84] and point to the importance of this subject. 

Much better measuring devices have been used in experimental and clinical studies. However, these devices are quite sensitive and require certain circumstances and settings to function properly. Devices that are to be used in realistic environments must be adjusted accordingly and are often reduced in their recording capacity. Future studies should consider the best possible measurement device, but also prioritize an environment as naturalistic as possible to maintain a semi-controlled but ecological study design.

## 5. Conclusions

As expected, this study observed that teachers exhibit vocal demand responses in Lombard speech in realistic noisy environments. Vocal effort was found to be quite high which can lead to vocal fatigue in the long term. The findings emphasize the need for further studies investigating occupational vocal demand responses in order to develop individual prevention and treatment methods. To this end, voice accumulation in work-related situations can provide evidence-based responses to determine safe and healthy performance to meet vocal demands [8]. The results provide evidence for an association between ambient noise and vocal demand response, which has been found to cause the development of vocal symptoms [7]. It is therefore highly recommended that voice training be provided for teachers to maintain their vocal health. To provide optimal listening conditions for the students, it is important to create good acoustical circumstances and equip individuals with the knowledge and ability to improve voice ergonomics [1,85] and to retain clear articulation.

## Figures and Tables

**Table 1:** Mean values (with standard deviations) of the measured parameters by condition.

	Quiet Condition	Noisy Condition	Statistics
**Fundamental Frequency (in Hz)**	Female: 220.7 (28.1)	Female: 286.4 (50.9)	t = −7.095; *p* < 0.001
	Male: 142.5 (13.8)	Male: 185,4 (29.9)	t = −3.595; *p* < 0.001
**Voice SPL (in dB(A))**	76.3 (2.3)	84.6 (2.2)	t = −13.464; *p* < 0.001
**Noise SPL (in dB(A))**	57.4 (4.9)	68.8 (4.8)	t = −9.758; *p* < 0.001
**Phonation time (in percent)**	60.7 (7.4)	71.6 (5.6)	t = −6.550; *p* < 0.001

**Table 2:** Occurrences of short pauses during reading in number and percentage by condition.

Pause Duration	Quiet Condition	Noisy Condition
**100 ms**	266 (81.3%)	167 (74.2%)
**200 ms**	61 (18.7%)	58 (25.8%)
**Total**	327 (100%)	225 (100%)

**Table 3:** Vocal demand responses in noisy environments, the resulting vocal risk and recommendations to maintain vocal health.

Voice Aspect	Response	Vocal Risk	Recommendation
**Voice SPL**	Increases	Increase in subglottal pressure and	Increase only as high as necessary for maintaining communication and decrease when not necessary
		Mechanical stress on vocal folds	
**ƒ_o_**	Increases	increased number vocal fold vibrations, subglottic pressure and mechanical stress on vocal folds	Increase speaking pitch with caution and combine it with supportive resonance strategies
**Voicing duration**	Lengthen	increased number vocal fold vibrations	Use it effectively for sound production
**Inhalation duration**	Remain similar	With increased voicing leads to longer phonation on lower lung volume, related to higher laryngeal position and pressed phonation	Take time to inhale deeper
**Short pauses**	Shortens	Loss of articulation	Maintain clear articulation

## Data Availability

The datasets generated during the study are available from the corresponding author on request.
